# Systems analysis and improvement approach to optimize tuberculosis (SAIA-TB) screening, treatment, and prevention in South Africa: a stepped-wedge cluster randomized trial

**DOI:** 10.1186/s43058-024-00582-z

**Published:** 2024-04-16

**Authors:** Brittney J. van de Water, Meredith B. Brooks, Refiloe Matji, Betty Ncanywa, Freck Dikgale, Nadia N. Abuelezam, Bulelwa Mzileni, Miyakazi Nokwe, Singilizwe Moko, Lindiwe Mvusi, Marian Loveday, Sarah Gimbel

**Affiliations:** 1 Connell School of Nursing, Boston College, 140 Commonwealth Avenue, Chestnut Hill, MA 02467 USA; 2 School of Public Health, Boston University, 715 Albany Street, Boston, MA 02118 USA; 3 AQUITY Innovations, 114 Sovereign Drive, Centurion, South Africa; 4 AQUITY Innovations, Greenacres Park, Gqeberha, South Africa; 5 Department of Health, Sarah Baartman District, 16 Grace Street, Gqeberha, South Africa; 6 Department of Health, Eastern Cape, Dukumbana Building, Bisho, South Africa; 7 Walter Sisulu University, Mthatha, South Africa; 8 National Department of Health, 1112 Voortrekker Road, Pretoria, South Africa; 9 HIV and Other Infectious Diseases Research Unit, South African Medical Research Council, Francie Van Zijl Drive, Parow Valley, Cape Town, South Africa; 10 Department of Child, University of Washington, Family & Population Health Nursing, Gerberding HallSeattle, WA 98195 USA

**Keywords:** Capacity building, Implementation science, Infectious disease, Sub-Saharan Africa

## Abstract

**Background:**

The use of systems engineering tools, including the development and use of care cascades using routinely collected data, process mapping, and continuous quality improvement, is used for frontline healthcare workers to devise systems level change. South Africa experiences high rates of tuberculosis (TB) infection and disease as well as HIV co-infection. The Department of Health has made significant gains in HIV services over the last two decades, reaching their set “90–90-90” targets for HIV. However, TB services, although robust, have lagged in comparison for both disease and infection. The Systems Analysis and Improvement Approach (SAIA) is a five-step implementation science method, drawn from systems engineering, to identify, define, and implement workflow modifications using cascade analysis, process mapping, and repeated quality improvement cycles within healthcare facilities.

**Methods:**

This stepped-wedge cluster randomized trial will evaluate the effectiveness of SAIA on TB (SAIA-TB) cascade optimization for patients with TB and high-risk contacts across 16 clinics in four local municipalities in the Sarah Baartman district, Eastern Cape, South Africa. We hypothesize that SAIA-TB implementation will lead to a 20% increase in each of: TB screening, TB preventive treatment initiation, and TB disease treatment initiation during the 18-month intervention period. Focus group discussions and key informant interviews with clinic staff will also be conducted to determine drivers of implementation variability across clinics.

**Discussion:**

This study has the potential to improve TB screening, treatment initiation, and completion for both active disease and preventive measures among individuals with and without HIV in a high burden setting. SAIA-TB provides frontline health care workers with a systems-level view of their care delivery system with the aim of sustainable systems-level improvements.

**Trial registration:**

Clinicaltrials.gov, NCT06314386. Registered 18 March 2024, https://clinicaltrials.gov/study/NCT06314386. NCT06314386.

**Supplementary Information:**

The online version contains supplementary material available at 10.1186/s43058-024-00582-z.

Contributions to the literature

- Our study examines if delivery of a user-friendly, low-cost package of systems engineering tools co-delivered iteratively by study personnel and district managers in a low-resourced, public sector health system improves health system performance and patient outcomes.
- Research on systems engineering tools has largely come from high-resourced health systems, with little data from low- and middle-income countries
- This is the first trial of the systems analysis and improvement approach (SAIA) for TB in a low-resource, high-burden setting.

## Background

Tuberculosis (TB) has been a leading infectious disease killer globally for decades despite the availability of robust diagnostics, effective prevention, and treatment [1]. One challenge has been poor implementation of comprehensive TB programs in low-resource settings where the majority of the global TB burden lies [2–12]. Additionally, one billion people are estimated to be infected with TB globally, with 5–15% of people at risk of becoming sick with TB disease [1]. Therefore, tackling TB systematically clinic by clinic and at the community level is important. Molecular testing and whole-genome sequencing have shown that in high-burden settings, the majority of household contacts have unmatched sensitivity patterns to the index patient in their household, thus inferring they were not infected in the household, but rather in a community setting [13–16]. So, although household contacts are a known high-risk group to screen for TB, using innovative methods to engage and empower clinics and communities in screening measures among high-risk populations is key to close existing case-detection gaps [17–19]. People living with HIV (PLH) are an important high-risk population to focus efforts on, as HIV is a leading driver for TB and TB is the leading cause of death among PLH [1, 20].

HIV is a main driver of TB in South Africa, one of 30 high burden TB countries, with nearly 60% of people newly diagnosed with TB also co-infected with HIV [20, 21].The TB care cascade evaluates gaps in care along the sequential steps of care for individuals with TB [22]; in South Africa, losses have been measured at multiple steps: 5% at test access, 13% at diagnosis, 12% at treatment initiation, and 17% at successful treatment completion [23]. These individual “gaps” in the cascade mount up to a 57% loss across the full TB care cascade in South Africa. The inequity in gaps is more stark among individuals coinfected with HIV and especially among individuals with drug-resistant TB with an overall 88% loss for those with drug-resistant TB [23]. Increasing the proportion of individuals eligible to complete each step of the care cascade is essential to reduce the TB burden, as those individuals who go undiagnosed and untreated contribute to further TB transmission and many of those diagnosed failing to complete the cascade [1, 24].

Even less is known about the care cascade for TB prevention treatment (TPT) [25, 26]. An estimated 80% of South Africans are infected with TB [21, 27]. This has created a vast reservoir of people at risk of progressing to TB disease, especially recent contacts of TB patients and PLH [28, 29]. Therefore, it is imperative to screen and treat not only TB disease but also subclinical TB infection in this high burden setting [30]. This is critical as South Africa recently updated their TPT guidelines, expanding eligibility to all household contacts in late 2021 [31]. Understanding how guidelines can be optimized is critical to implementation success and sustainability [32]. A global meta-analysis of the TPT cascade from 2016 revealed that of individuals intended for TB screening, less than 19% successfully completed TPT, indicating an opportunity to leverage strategies to close gaps in TB care [25].

There is growing international recognition for interventions to end the TB epidemic; gaining political will, community acceptance, avoiding stigma, and emphasizing feasibility of eliminating TB are all critical [33, 34]. Passive case finding (PCF)—the notion of waiting until individuals become symptomatic with TB disease to report to the health system—is the most common form of case finding. However, this is insufficient to reduce TB incidence and prevalence. Rather, a systematic and comprehensive active case finding (ACF) strategy must be employed where programs search for and screen high-risk groups to detect disease at an earlier stage, ideally before further transmission [35]. Recently the WHO Asia–Pacific region published ACF optimization implementation lessons and evidence for achieving TB elimination, including 17 studies assessing community- and household-based TB programs focused on detection, prevention, and treatment [36]. These, along with other historical examples of TB elimination in Alaska, Greenland, and Australia, provide necessary examples of scalable implementation efforts [37–40].

The Systems Analysis and Improvement Approach (SAIA) is an evidence-based implementation strategy designed to optimize cascade performance, is feasible for frontline healthcare workers and managers, and may be applicable to optimize TB care [41]. We aim to adapt SAIA for TB (SAIA-TB), expanding upon successful SAIA adaptations that have been trialed across a range of clinical settings, in sub-Saharan Africa and the USA, and leverage preliminary TB cascade data collected in this setting [4, 5, 41–47]. Preliminary work developing the SAIA-TB cascade by the investigative team has identified six routinely collected TB data points to serve as linked steps across TB services (screening, evaluation, diagnosis, linkage to care, treatment completion, and TB-free survival). The resultant TB cascade analysis tool (TCAT) will be refined as part of the SAIA-TB implementation strategy to aid frontline healthcare workers and managers to optimize cascade performance. In addition to the TCAT which is specifically tailored to the TB care cascade in our proposed study setting, SAIA-TB will also employ monthly process flow mapping and continuous quality improvement cycles with clinic-led discussions to implement clinic-identified micro-interventions to optimize the TB care delivery for patients as each of these components are core to the SAIA strategy [48].

### Goals and objectives

In this study, we will (1) evaluate the effectiveness of SAIA-TB use in clinics on TB cascade outcomes for TB patients and with high-risk contacts (specifically among PLH) and (2) determine the drivers of SAIA-TB implementation success or failure across clinics.

## Methods

### Description of the SAIA-TB implementation strategy

**Fig. 1:**
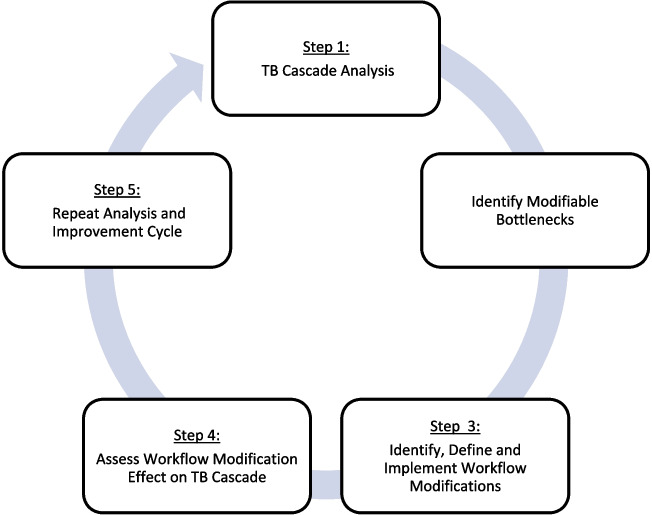
Adapted systems analysis and improvement approach cycle for TB (SAIA-TB)

SAIA-TB is similar to previously described SAIA applications [44, 48, 49]. The facility-based, five-step iterative process is designed to guide clinic staff and managers in understanding and improving their TB services and optimizing MOH-defined evidence-based interventions, namely, TB screening, prevention, and treatment protocols in order to improve patient outcomes through clinic teams’ delivery of these protocols. The SAIA-TB implementation strategy uses tools borrowed from systems engineering to strengthen the care teams view of TB delivery at their facility—across the care cascade from TB screening to TB-free survival. Using routinely collected data and health systems knowledge, the tools guide clinic staff’s identification and prioritization of modifiable barriers, followed by continuous quality improvement cycles to implement and rapidly evaluate appropriate clinic-level solutions (Fig. 1). SAIA-TB includes the following steps.

#### Step 1

**Fig. 2:**
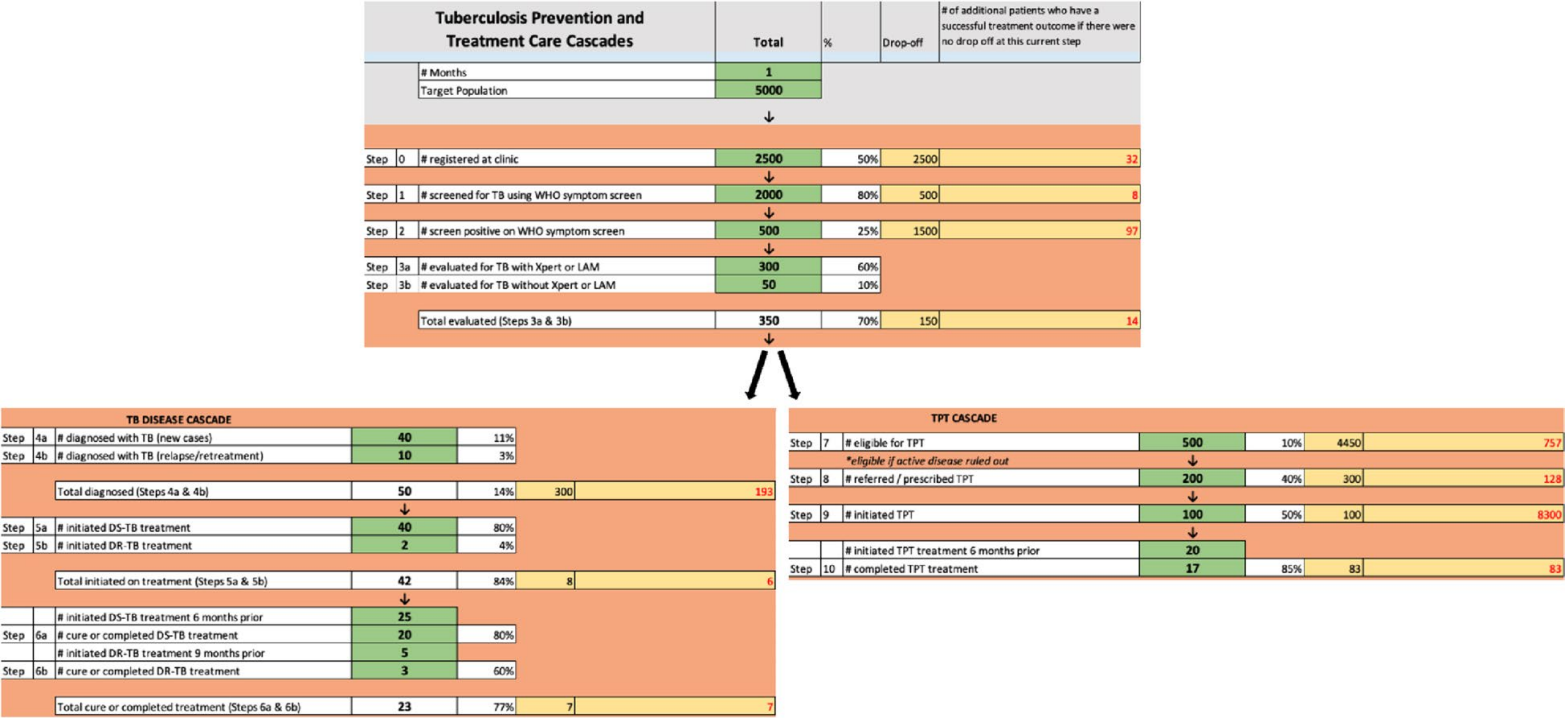
TB cascade analysis tool (TCAT)

Understand targeted TB cascade performance and identify priority areas for improvement. The TB cascade analysis tool (TCAT) (Fig. 2) uses routine data to provide a rapid, systems-level view of drop-offs along the TB cascade for outpatient clients and priority sub-populations (PLH, children under 5 years, and household contacts), with an optimization function allowing the user (i.e., clinic staff) to rapidly assess how many additional people will be served if only one step is fully optimized while other steps stay the same [42]. As an analytic tool, TCATs help frontline staff and clinic managers to prioritize where to intervene by providing a view of the greatest potential for flow improvements across the entire cascade. This tool calculates the number and proportion of individuals flowing through each step of the TB cascade, broken down into the flow from screening through treatment completion.

#### Step 2

Process mapping to identify clinic-level modifiable bottlenecks in TB management. Enabling facility-level staff to identify and gain consensus on bottlenecks to address in their TB systems essential to defining innovations to implement. SAIA-TB applies sequential patient process flow mapping procedures, along with workflow observation, to identify bottlenecks and guide discussion on opportunities for modifications across the care team [50, 51]. Study personnel will work with clinic staff, across all services and staff levels, to map the existing pathways patients with TB and high-risk individuals (PLH, children under 5 years, and household contacts) take at their facility.

#### Step 3

Define and implement clinic-specific workflow adaptations to address modifiable bottlenecks. After identifying modifiable barriers within cascade steps, clinic staff identify a simple, specific change to improve performance within the targeted step. Selected workflow adaptations should be feasible to implement, be within the scope of influence of clinic management and frontline staff, and be expected to lead to rapid, substantial improvements in the targeted cascade step. Ideas for adaptations will arise from brainstorming solutions with clinic staff, complemented by best practices from the literature and high performing services in South Africa (i.e., HIV services) and other TB programs [22, 49]. An implementation plan for the innovation is described in writing by clinic and study personnel to ensure consensus among clinic staff, and to clarify operational design and roles, including a future state process map that reflects processes after the modification. After defining the adaptation to be implemented, clinic staff will implement the proposed changes.

#### Step 4

Monitor changes in routine performance and initiate additional iterations. Clinic staff monitor change in routinely reported data from the TB cascade step selected for improvement. Measuring the absolute change in the proportion of patients progressing through targeted steps captures large, rapid improvements accompanying modifications.

#### Step 5

Repeat cycle. Systems engineering improvement processes are by definition iterative, with ongoing testing of innovations responsive to evolving, contextually specific barriers. Clinic staff will repeat steps 1 through 5 at the end of each cycle to identify novel approaches to modify previously identified barriers or, if the first cycle was successful, focus on improving priority bottlenecks identified [44, 49]. Steps 3 through 5 are analogous to continuous quality improvement [44, 49].

### SAIA-TB trial design

**Fig. 3:**
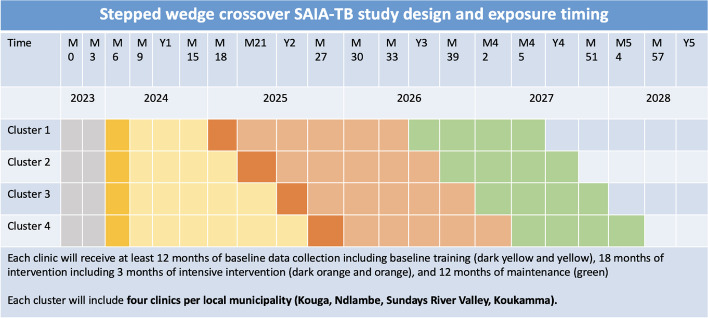
SAIA-TB study design and study timeline

This study is a 5-year, four-wave stepped wedge cluster randomized trial design to assess the effectiveness of the SAIA-TB strategy (Fig. 1; Consort Checklist, Additional file 1). Each wave includes four local municipalities within a district, with four health facilities per local municipality purposively selected to receive SAIA for a total of 16 facilities. Each local municipality represents a cluster and will be randomized for intervention wave. Each facility will have at least 12 months of baseline data collection followed by 18 months of intervention and 12 months of a measured maintenance phase (Fig. 3).

### Process for introducing SAIA-TB

#### Pre-implementation and baseline data collection

**Fig. 4:**
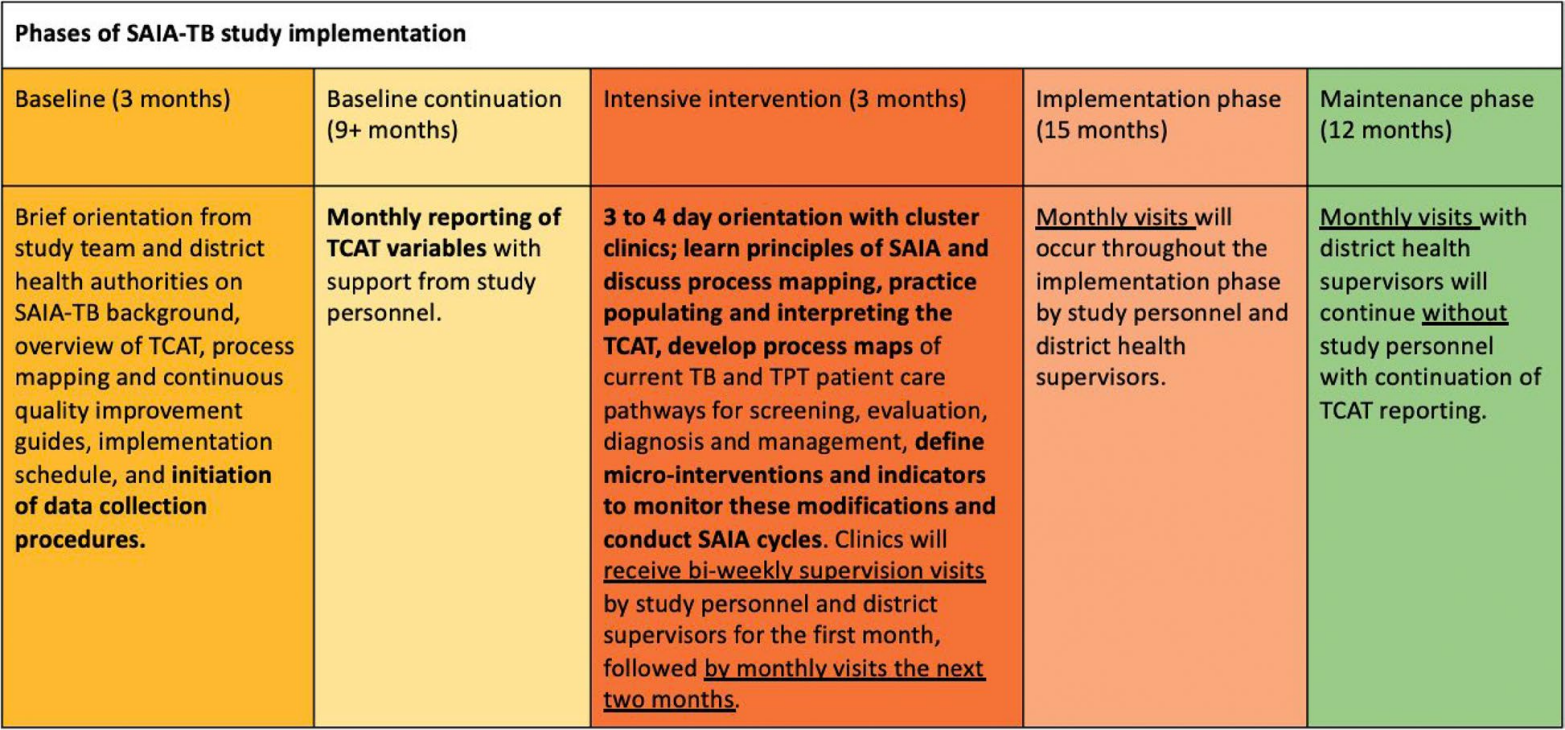
Phases of SAIA-TB study implementation

SAIA-TB’s standard operating procedures (SOPs), including the delivery and training schedules and intervention guides and tools (TCAT, process mapping, and continuous quality improvement guides), will be refined from in preparation for the SAIA-TB trial. In the 12 months prior to SAIA-TB implementation, the study team will engage in *baseline data collection* to ensure routine data points needed to populate the TCAT are collected reliably and are available at all facilities (Fig. 4). The project manager (a professional nurse) will conduct a baseline facility-level assessment (described below) and introduce the study to facility teams with a brief orientation on SAIA-TB background and SAIA main components (including an overview of the TCAT, process mapping, and continuous quality improvement guides), as well as share the implementation schedule, and begin data collection procedures. Baseline data collection will continue for at least 12 months in all clinics with monthly reporting of TCAT variables with support from study personnel.

#### Intervention phase

Clusters will begin the *intervention phase* in waves, starting every 3 months. Each cluster will receive a 3- to 4-day orientation from the study team and district health authorities on SAIA-TB components. Orientation will include practice with populating and interpreting the TCAT, developing process maps of current TB and TPT patient care pathways for screening, evaluation, diagnosis, and management, and engagement in their first CQI meeting whereby they identify, prioritize, and define their first micro intervention. Subsequently, district TB supervisors, together with study personnel, introduce SAIA-TB to intervention health facilities over a 2-day period. Within each local municipality, SAIA-TB is introduced to all facilities together, and then independent facility visits will be conducted. Facility teams receive bi-weekly supervision visits by study personnel and district supervisors for the first month, followed by monthly visits throughout the remainder of the 18-month intervention phase, consistent with most adaptations of SAIA globally.

#### Maintenance phase

During the final 12-month period, SAIA-TB will have a *maintenance phase*, whereby monthly visits will continue by district authorities without study personnel to evaluate SAIA-TB’s sustainability with moderate resource investment.

It is expected that SAIA cycles will continue to occur monthly, with an average of 12 SAIA cycles per year per facility. A SAIA core component that will be maintained in SAIA-TB is provision of a flexible clinic support fund to address equipment needs for TB management (scales, some transportation costs for patients, etc.) as well as to support smaller workflow modifications, which will continue throughout both the intervention and maintenance periods. During monthly visits, facility staff participating in the intervention will complete questionnaires measuring drivers of SAIA-TB and participate in facilitation of process mapping, supportive clinical consultations, and systems engineering discussions.

### Study setting and eligibility criteria

#### Study setting

The Eastern Cape has the highest burden of TB nationally, with a TB incidence of 692 per 100,000 people, and Sarah Baartman district has the highest incidence in the country with 1022 cases per 100,000 [21]. The co-infection TB/HIV rate is 41% among patients with drug-sensitive TB and 62% among those with drug-resistant TB in the Eastern Cape [52]. The Sarah Baartman district has an average household size of 3.42 people per house and 9% of individuals living in informal dwellings. Over 77% of individuals have access to flush toilets, with 58% being female headed households and 6% being child headed households. There is a youth unemployment rate of 31% for individuals 15 to 34 years and an overall unemployment rate of 25% [52].

#### Eligibility criteria

**Fig. 5:**
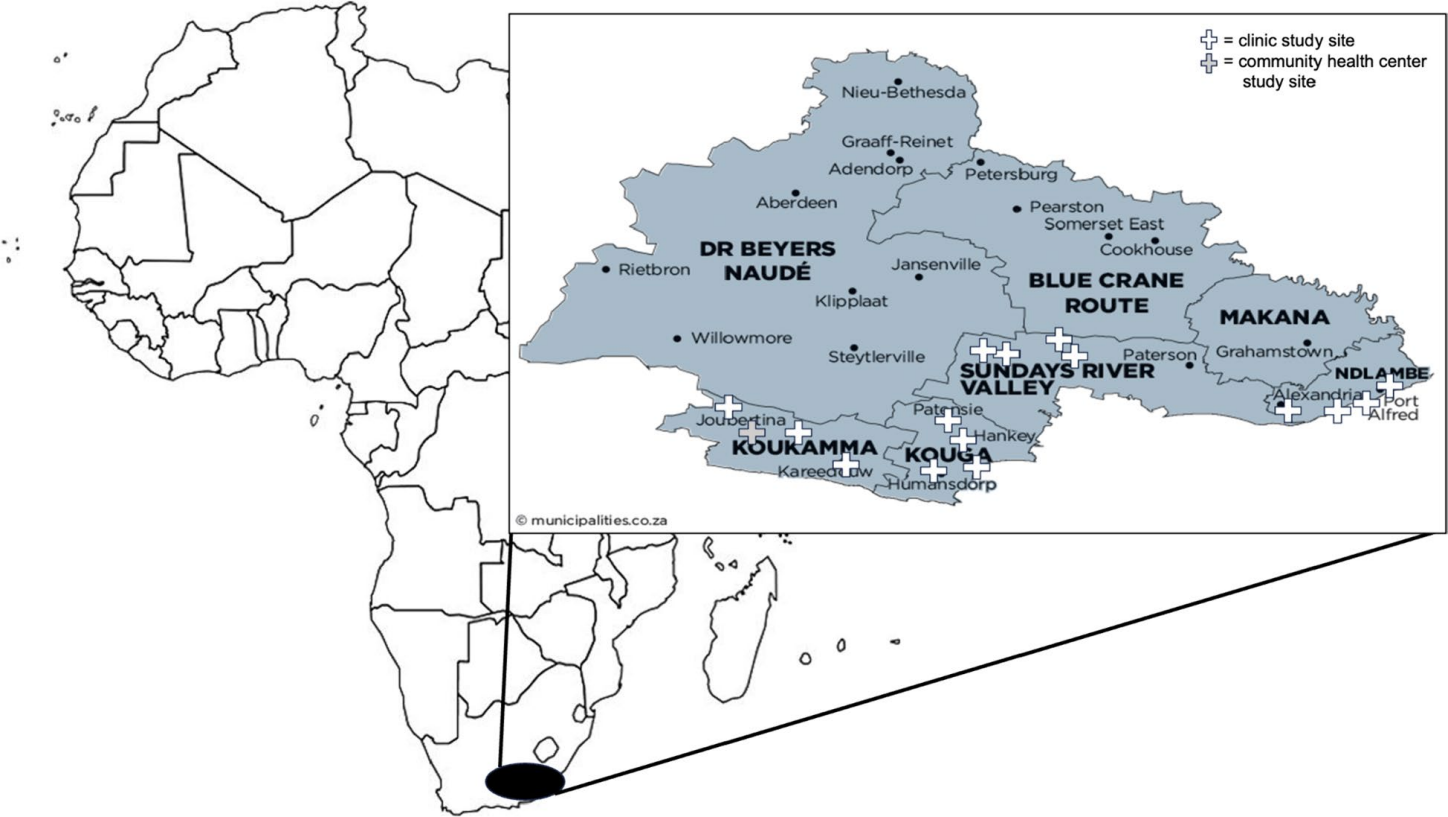
Africa, with Eastern Cape, South Africa, highlighted and Sarah Baartman district map with study locations

This study will take place in four local municipalities within the Sarah Baartman district, Eastern Cape, South Africa (Fig. 5). Services for TB across South Africa are primarily provided through the public sector, and all proposed study clinics are public. Study clinics are evenly split across four local municipalities in Sarah Baartman district—Ndlambe, Sundays River Valley, Kou-Kamma, and Kouga (Table 1). These local municipalities were chosen in partnership with district of health officials for their heterogeneity in populations, proximity to Gqeberha (within 2 h’ drive to where the study office is located), patient volume (at least 4500 outpatient visits in 2022), rurality (mix of rural, semi-rural, semi-urban), and staffing mixture. Each clinic has at least two professional nurses, one or two pharmacy technicians, a data clerk, and a social worker, as well as multiple community health workers who are clinic-based or part of ward-based outreach teams.

**Table 1:** Sarah Baartman district statistics including area, population density, and population by local municipality

	Sarah Baartman District	Dr Beyers Naude LM	Blue Crane Route LM	Makana LM	Ndlambe LM	Sundays River Valley LM	Kouga LM	Kou-Kamma LM
Area (square km)	58,245 km^2^	28,653 km^2^	11,068 km^2^	4376 km^2^	1841 km^2^	5995 km^2^	2670 km^2^	3642 km^2^
Population density (no)	8	3	3	17	38	10	45	13
Population (no)	483,024	77,748	30,414	74,729	69,130	62,306	120,273	48,424

### Randomization

Clusters will be randomized without restriction into implementation waves during a meeting with provincial and district health authorities and study leadership in Eastern Cape in 2024 prior to the intervention phase. Once allocated, local municipalities will not be informed of the implementation timeframe for their wave. To limit contamination between intervention sites and sites not yet receiving the intervention, all clinics within the same local municipality will be allocated to the same cluster.

## SAIA-TB impact assessment

### Study population

All patients accessing outpatient department care services at study facilities during the study period, including those screened for TB care (TB preventive treatment or disease treatment) as part of outreach events off-site or diagnosed with TB at referral hospitals and are down-referred back to their catchment clinic, are included.

### Exposure definition

Facilities will be considered unexposed prior to the initiation of the intervention in their local municipality and exposed thereafter. Individuals’ exposure to the intervention will be based on the exposure status of the facility in the calendar month in which they first entered TB care (including those newly identified as eligible for TPT and TB treatment and those already on TPT or TB treatment).

### Outcomes

We will measure each indicator across the TB care cascade from TB screening to TB-free survival. (Table 2). Primary outcomes include the mean change in proportion of individuals (1) screened for TB disease, (2) initiated on TPT, or (3) initiated on TB disease treatment combined across all facilities during the intervention period (exposed) compared to baseline (unexposed). Primary outcomes were selected based on sensitivity to system-level improvements; they represent SAIA-TB steps that, if changed, would meaningfully alter patterns of TB-related morbidity and they are routinely collected. They are relatively easy to collect and readily understood by clinic managers and frontline staff. Indicators will be assessed monthly over the study period (including 12 months baseline, 18 months intervention, and 12 months maintenance phase).

**Table 2:** SAIA-TB primary and secondary outcomes

SAIA-TB primary and secondary outcome variables	
**TB indicator (variable)**	**Numerator/denominator**
TB screening^a^	*#screened with WHO symptom screen* # target population
TB evaluation	*# receiving a diagnostic evaluation (GXP/LAM)* # positive screening
TB diagnosis	*# diagnosed with TB (GXP/LAM/clinical)* # who completed a diagnostic evaluation
Linkage to care for TPT^a^	*# initiating TPT* # negative diagnostic evaluation + eligible for TPT
Linkage to care for TB disease^a^	*# initiating TB treatment* # positive diagnostic evaluation
Successful TPT outcome	*# completing TPT* # initiating TPT
Successful TB outcome^b^	*# cure or complete treatment for TB disease* # initiating TB treatment
TB-free survival	*# TB-free (CXR/GXP) 12 months* *after initiating TPT or TB disease treatment* # successful outcomes for TPT or TB disease

*CXR* Chest x-ray, *GXP* GeneXpert, *LAM,* Urine lipoarabinomannan

^a^Primary outcome variable

^b^WHO definition

Secondary outcomes include the additional TB cascade indicators (TB evaluation, diagnosis, treatment outcome, and TB-free survival); however, some of these are more dependent on a mix of individual-level factors (i.e., adherence to medication) (Table 2). These indicators are clinically important and can be affected at a system-level. Outcomes will be assessed for all patients receiving care, as well as assessed by important sub-groups, such as by HIV status, age (under 5 years), and local municipality (cluster).

### Data sources

Patient-level TB data will be sourced from existing programmatic Ministry of Health facility registries and patient-level forms. Slight modifications may need to be designed to capture the entire TB care cascade from screening to TB-free survival. Data will be abstracted from these existing paper registries and patient forms at least weekly by study personnel into a study database via REDCap on tablet or laptop [53]. As part of routine care, each patient with TB is assigned a unique identification number that links across service points and clinics, which will be used to abstract registry data for study outcome measures. The database will generate on-demand reports with monthly indicators to populate the TCAT.

### Power and sample size

Power calculations are based on the primary outcome: mean change in proportion of individuals who complete screening for TB. TB screening was selected as the primary outcome as existing South African guidelines call for TB screening to be routinely implemented in outpatient services, and it is where the largest gap in the TB care cascade is currently. In a stepped wedge study, exposed and unexposed observation periods take the place of “arms” in parallel cluster trials. Outcomes (TB screening, TPT initiation, and TB treatment initiation) at baseline have been estimated to be 39% of eligible individuals screened for TB, 31% eligible individuals initiating TPT, and 70% eligible individuals initiating TB treatment per preliminary and national data (unexposed) [5, 23, 25]. We need a sample size of 978 individuals screened per cluster per time period to have 99% power (alpha = 0.05, and intracluster correlation of 0.01) to detect down to a 5% difference of proportion. Therefore, each cluster, over the course of all 5 time periods, needs to screen 4890 individuals. With four clusters, that will produce a total sample size of 19,560. Power calculations were completed using the Pass power analysis and sample size statistical software [54].

### Data analysis

Intervention analysis for primary outcomes will include generalized linear mixed modeling comparing study outcomes between exposed and unexposed times for outcomes described in Table 2 [31, 55]. Models will account for clustering by local municipality and will assess the impact of adjustment for patient-level covariates (i.e., age, sex, HIV status, and comorbidities) as well as clinic-level covariates (i.e., patient volume, staffing levels) and time (months since study initiation). We will assess missing data for randomness and employ multiple imputation, if possible [56, 57]. Additionally, we will assess outcomes among all individuals, including patients lost to follow-up, following an intent-to-treat principle [58–60]. Secondary analyses will test for interaction between exposure and time (temporal and time-to-exposure) to avoid a bias estimate of treatment effect [31]. We will also conduct a controlled, segmented time-series analysis incorporating monthly clinic-level estimates from the entire study period (segmented into 12-month baseline, 18-month intervention, and 12-month maintenance phases). This secondary analysis will allow us to fully use available data to assess intervention impact, address both serial and intra-class correlation, and assess temporal patterns in study outcomes [55, 58].

### Determining drivers of SAIA-TB intervention implementation heterogeneity

We will apply four implementation science theories, models, and frameworks to examine the implementation process, focusing on clinics as the organizational level.

#### Organizational Readiness for Implementing Change

Organizational Readiness for Implementing Change (ORIC) assesses the extent to which organizational members are psychologically and behaviorally prepared to implement organizational change, affecting decisions to adopt implementation strategies such as SAIA-TB [61, 62]. Readiness includes (1) change commitment, a shared resolve to implement a change, and (2) change efficacy, a shared belief in the collective capability to implement a change. Therefore, to understand readiness for adopting SAIA-TB, we will apply the ORIC assessment scale to at least 6 health workers per study facility (*n* = 48) and district and provincial management (*n* = 12). Analysis will test whether sufficient inter-rater reliability and inter-rater agreement exist to aggregate individual responses to the facility level; if tests do not justify aggregation, we will use a measure of intra-facility variability in readiness rather than a facility-level mean in analysis. This will provide readiness profiles for each facility as they initiate implementation, complementing adoption, implementation, and effectiveness data in understanding the broad impact of SAIA-TB. The ORIC assessments have been used in many SAIA settings as well as in other contexts by the study team and in South Africa [63–65].

#### Facility-level structural readiness assessments

Standardized readiness assessments, adapted and abbreviated from the service availability and readiness assessment (SARA) tool, will be carried out immediately prior to each phase (baseline, intervention, maintenance) in all 16 study facilities to assess structural readiness to deliver TB services (staffing levels, attributes and training, availability of essential commodities, equipment, supplies, and clinic infrastructure) [66]. This adapted tool has been piloted in a neighboring Eastern Cape district for TB services by the study team. These facility-level assessments will help inform what drove implementation success or failure and to examine if structural changes took place during the study period beyond TB services.

#### Consolidated Framework for Implementation Research

We will use the Consolidated Framework for Implementation Research (CFIR) to guide an in-depth examination of the implementation process, define SAIA-TB core components, and describe determinants of implementation success and failure across implementing local municipalities and facilities [67]. The study team has experience in using CFIR for TB in the South African context, and in prior SAIA trials, and discussion guides will be developed using available CFIR tools to gather data about constructs from the five CFIR domains plus an added systems domain used in low-resource settings [44, 68, 69]. After the completion of the intervention phase, and receiving written consent from participants, we will conduct focus group discussions (FGDs) in each intervention facility with 6–8 clinic staff (sufficient to generate conversation without being too large to become intimidating) (*n* = 96–128) participating in SAIA-TB [70]. Facilities will be classified as either high or low performing (identified by facilities’ fidelity to the SAIA-TB intervention, defined as the number and frequency of SAIA-TB cycles conducted, and the consistent use of quantitative CQI data to inform progress). Focus group discussions will be purposively held by individual facilities in order to classify them as high and low implementation fidelity and to uncover salient features of successful implementation. In-depth interviews (IDIs) will also be conducted with facility (*n* = 16) and district and provincial (*n* = 12) managers participating in SAIA-TB. These IDIs will allow for the collection of potentially sensitive information from leadership that might otherwise might not be shared in a larger group, and to give lower-ranking team members more freedom to speak freely in the FGDs, regarding leadership engagement or organizational culture or hierarchy. The IDIs allow for the capture of individual experience with disseminating and implementing SAIA-TB and capture intervention adaptations over time, such as staff attitudes or identification with the organization. Participants will be purposively selected for a balance of individuals across service locations and roles within the facilities (i.e., nursing, pharmacy, reception, social work, community health workers). We will repeat an equal number of FGDs and IDIs at the end of the maintenance phase to explore the implementation process and adaption that occurred during that period. The same sampling scheme will be used.

Interviews (FGDs and IDIs) will be conducted in English (the working language across South Africa) by an experienced facilitator, audio-recorded, and transcribed verbatim. Focus groups will also have a note taker present. Two coders in a stepwise, iterative process will code transcripts and conduct content analysis within a deductive framework to identify key implementation themes using selected CFIR constructs, yet allowing flexibility for additional themes to emerge. Coding will be compared across pairs and differences discussed prior to final coding. Case memos will be documented and three analysts will assign ratings for each construct. Ratings will reflect the positive or negative influence (valence) and strength of each construct, as previous applications of CFIR have shown [43, 71]. Finally, constructs will be coded as missing too much data (M), not (0), weakly (+ 1/ − 1), or strongly (+ 2/ − 2) distinguishing low/high performance. These qualitative findings will be used to develop recommendations for SAIA-TB implementation, specific to its core components, intervention adaptions, and lessons learned.

#### Implementation fidelity

We will prospectively document fidelity to the protocol and track implementation using surveys filled out by study nurses and study data clerks. We will capture the following: number of planning sessions each facility conducted, number of participants in sessions, number of workflow modifications tested, and the content and results of modifications.

#### Maintenance

We will measure the extent to which SAIA-TB is maintained over time at the organizational (i.e., facility) level. This will be measured as the proportion of facilities continuing to implement the intervention as designed. We will describe the proportion of local municipalities (clusters) and facilities continuing to implement SAIA-TB at 6 and 12 months post-intervention (target: > 90% at 6 months, > 75% at 12 months). Continued maintenance is defined as holding monthly SAIA-TB meetings. We will also evaluate maintenance through in-depth interviews and focus group discussions with district and facility staff on perspectives of determinants of sustained implementation or barriers to maintenance at the end of the maintenance phase as described above.

### Ethics

Approval by the institutional review board at Boston College was obtained (23.139.01). Additionally, approval was provided by the South African Medical Research Council (EC012-5/2023). The study was registered with the Eastern Cape Department of Health National Health Research Database (September 2023). This study was also registered with ClinicaTrials.gov, registration number NCT06314386.

### Study status

Implementation of the SAIA-TB intervention began in October 2023 with baseline facility assessments. At the time of submission, baseline data collection is occurring in all 16 clinics and the intense intervention phase is planned to start in January 2025.

## Discussion

At the completion of this pragmatic stepped wedge cluster randomized trial, important knowledge regarding the effectiveness of SAIA-TB on TB cascade optimization for patients with TB and high-risk contacts, specifically PLH in a high burden TB/HIV context will be gained. Drivers of SAIA-TB implementation variability across clinics and fidelity to the intervention will be better understood, providing robust evidence for translation, scale-up, and dissemination. Given the rapidly evolving landscape of TB guideline development on the global and national level, this intervention provides opportunity to be at the forefront of implementing newly released TPT regimens as they become available within the public sector. Our agility within the SAIA strategy provides clinics opportunity for knowledge sharing and innovation as we take lessons learned from the pandemic and translate and harness them for TB and HIV services [72, 73]. Finally, adaptations from previous SAIA iterations will be documented to aid in future SAIA implementation in additional health conditions and across geographically diverse settings.

### Supplementary Information

**Supplementary Material 1.**

## Data Availability

Not applicable.

## Abbreviations

CFIR: Consolidated Framework for Implementation Research
CQI: Continuous quality improvement
HIV: Human immunodeficiency virus
ORIC: Organizational Readiness for Implementing Change
SAIA: Systems analysis and improvement approach
TB: Tuberculosis
TCAT: Tuberculosis cascade analysis tool
WHO: World Health Organization
